# Mechanisms of Innate Immune Sensing of HTLV-1 and Viral Immune Evasion

**DOI:** 10.3390/pathogens12050735

**Published:** 2023-05-19

**Authors:** Suchitra Mohanty, Edward W. Harhaj

**Affiliations:** Department of Microbiology and Immunology, Penn State College of Medicine, Hershey, PA 17033, USA; ewh110@psu.edu

**Keywords:** HTLV-1, ATLL, immune sensors, restriction factors, immune evasion, Tax

## Abstract

Human T lymphotropic virus-1 (HTLV-1) was the first identified oncoretrovirus, which infects and establishes a persistent infection in approximately 10–20 million people worldwide. Although only ~5% of infected individuals develop pathologies such as adult T-cell leukemia/lymphoma (ATLL) or a neuroinflammatory disorder termed HTLV-1-asssociated myelopathy/tropical spastic paraparesis (HAM/TSP), asymptomatic carriers are more susceptible to opportunistic infections. Furthermore, ATLL patients are severely immunosuppressed and prone to other malignancies and other infections. The HTLV-1 replication cycle provides ligands, mainly nucleic acids (RNA, RNA/DNA intermediates, ssDNA intermediates, and dsDNA), that are sensed by different pattern recognition receptors (PRRs) to trigger immune responses. However, the mechanisms of innate immune detection and immune responses to HTLV-1 infection are not well understood. In this review, we highlight the functional roles of different immune sensors in recognizing HTLV-1 infection in multiple cell types and the antiviral roles of host restriction factors in limiting persistent infection of HTLV-1. We also provide a comprehensive overview of intricate strategies employed by HTLV-1 to subvert the host innate immune response that may contribute to the development of HTLV-1-associated diseases. A more detailed understanding of HTLV-1-host pathogen interactions may inform novel strategies for HTLV-1 antivirals, vaccines, and treatments for ATLL or HAM/TSP.

## 1. Introduction

The human T lymphotropic virus type 1 (HTLV-1) was discovered as the first human oncogenic retrovirus and belongs to the family Retroviridae and genus Deltaretrovirus [1,2]. HTLV-1 is an enveloped complex retrovirus and the causative agent of an aggressive neoplasm of mature CD4+CD25+ T-cells known as adult T-cell leukemia/lymphoma (ATLL) [3]. HTLV-1 is also associated with several inflammatory disorders such as HTLV-1-associated Myelopathy/Tropical Spastic Paraparesis (HAM/TSP), uveitis, infectious dermatitis, and polymyositis because of dysregulated immune responses [4,5]. Vertical transmission (i.e., breast feeding from mother to infant) is the primary mode responsible for the spread of HTLV-1 infection, and thus contributes to clustered HTLV-1 infection foci in specific geographical areas of the world including southern Japan, the Caribbean basin, sub-Saharan Africa, parts of the Middle East (Iran), South America (in particular Brazil, Colombia, Chile, and Peru), and indigenous communities in central Australia (e.g., Alice Springs) [6,7,8]. There are an estimated 10 million asymptomatic HTLV-1-infected individuals worldwide, yet only a low percentage (2–5%) of infected individuals develop HTLV-1-associated diseases after decades of latency. It is now well established that disease progression is tightly correlated with HTLV-1 viral burden or proviral load in infected individuals [9,10]. Likewise, horizontal transmission through intravenous drug injection, or blood transfusion, specifically in immunocompromised individuals, shortens the latency period and leads to the development of aggressive HTLV-1-associated pathogenesis [11]. ATLL (specifically acute or lymphoma type) is characterized as a highly aggressive and fatal malignancy with dismal prognosis and poor survival, typically between 6–12 months [12]. Thus, the development of antiviral therapeutics is critically important to prevent productive and chronic infection and to limit clinical symptoms to improve the survival of individuals with HTLV-1-associated diseases.

HTLV-1 is a highly oncogenic virus with regulatory proteins that can modulate host cellular signaling pathways and cell cycle checkpoints to enhance the clonal proliferation and long-term persistence of infected cells that can eventually lead to leukemia/lymphoma. In addition, HTLV-1 evades immune surveillance through limiting its replication via tight control of viral gene expression. Although HTLV-1 was discovered more than 40 years ago, the innate immune responses generated during the different stages of HTLV-1 infection remain poorly understood, likely due to the lack of a tractable infection model and unique infection mechanisms utilized by HTLV-1. However, there is accumulating research evidence describing immune escape strategies employed by HTLV-1 to counteract the host immune surveillance in infected individuals. In this review, we will discuss the current knowledge of innate immune sensing of HTLV-1 and the restriction factors involved to counteract viral replication at multiple levels. Additionally, this review also discusses the HTLV-1-mediated immune evasion strategies to prevent recognition and destruction of infected cells by the innate immune system.

## 2. HTLV-1 Genomic Organization and Viral Life Cycle

HTLV-1 is a spherical enveloped virus approximately 100 nm in diameter and contains linear, dimeric positive-sense and single-strand genomic RNA inside an envelope embedded with protruding viral glycoproteins (gp46 and gp21) [13]. The icosahedral capsid contains functional protease (pro), integrase (IN), and reverse transcriptase (RT) enzymes and a nucleocapsid with the viral genome. The HTLV-1 genome is approximately 9 kb with a 5′-cap and 3′ poly-A tail. Despite the small size of the genome, the viral genome contains features common to all retroviruses such as Gag, Pro, Pol and Env, and expresses multiple regulatory proteins through utilizing diverse strategies such as alternative mRNA splicing, polycistronic translation, frameshifting and antisense expression. The pX region, located in the 3′ end of the genome, encodes regulatory genes Tax, Rex, p21, p12, p13, and p30 through different open reading frames (ORFs). The viral genome is flanked at 5′ and 3′ ends with long terminal repeats (LTRs) crucial for viral transcription, integration, and translation [14].

The life cycle of HTLV-1 is analogous to other retroviruses; however, HTLV-1 cell-free virions are poorly infectious, and cell-to-cell spread between infected cells and target cells via the virological synapse is crucial for the transmission of the virus [15,16]. The virological synapse facilitates the transfer of the HTLV-1 core protein (Gag), and the viral RNA genome to uninfected cells. HTLV-1 can also infect cells through other mechanisms such as biofilm-like structures or tunneling nanotubes [17,18,19]. HTLV-1 entry into cells depends on sequential interactions of the viral envelope (Env) with heparan sulfate proteoglycans (HSPGs) followed by the formation of a complex with Neuropilin-1 (NRP1) and Glucose transporter-1 (GLUT1) leading to fusion of the viral membrane with the target cell plasma membrane [20,21,22]. After penetration into the target cell, the uncoating of the virus yields viral RNA in the cytoplasm of infected cells that undergoes reverse transcription to form double-stranded DNA for integration into the host genome as a provirus. The host polymerase II enzyme initiates viral transcription from the integrated provirus and generates viral mRNAs for translation into distinct viral proteins. The viral genomic RNA, along with the viral core proteins (Gag, Env, and Gag–Pol) are transported to the plasma membrane to form an immature viral particle with packaged viral RNAs during assembly. The capsid protein drives the formation of the Gag lattice in immature particles, following the interaction of p19 matrix (MA) and the inner leaflet of the cell membrane [23,24]. The budding viral particle undergoes protease-mediated maturation to form an infectious viral particle after release from the cell surface [25]. HTLV-1 preferentially infects CD4+ T lymphocytes in vivo and can also infect CD8+ T-cells, monocytes and dendritic cells (DCs), B cells, and astrocytes [26,27]. The infected myeloid cells were shown to produce HTLV-1 viral particles and transmit HTLV-1 to T-cells in vitro [27]. Thus, infected DCs may be critical in viral dissemination during primo-infection, and possibly, infected monocytes and pDCs could also play key roles in immune evasion during the chronic phase of infection and subsequent HTLV-1-associated diseases.

## 3. Immune Sensors of HTLV-1

**Figure 1:**
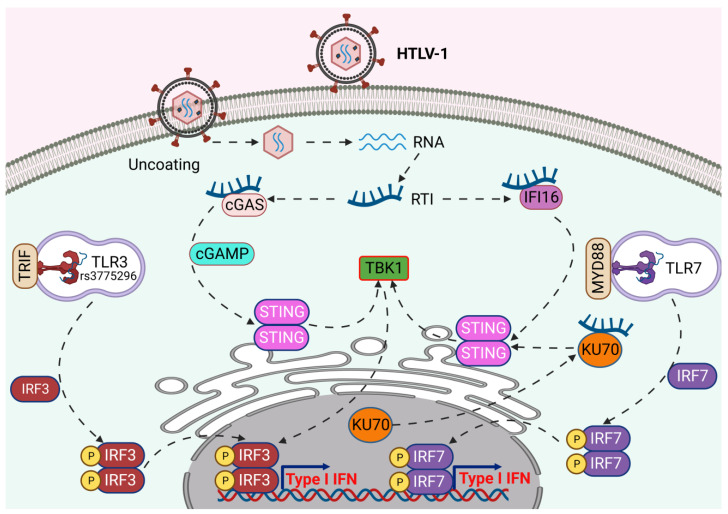
Immune sensing of HTLV-1 by pattern recognition receptors. Endosomal TLR3 and TLR7 recognize HTLV-1 RNA and initiate type I IFN production via IRF3 and IRF7-mediated immune signaling. The cytosolic sensors, cGAS-STING, IFI16, and Ku70 sense HTLV-1 reverse transcription intermediates (RTIs) for the activation of TBK1-mediated immune signaling to produce type I IFN. Created with BioRender.com.

Pathogen recognition receptors (PRRs) recognize pathogen-associated molecular patterns (PAMPs) in viruses, typically nucleic acids, to trigger an immune response through the secretion of type I IFN and proinflammatory cytokines [28]. PRRs are germline-encoded innate immune sensors localized either on the cell surface or within distinct intracellular compartments. The PRRs most relevant for virus infections include Toll-like receptors (TLRs), the retinoic acid-inducible gene I-like receptors (RLRs), the nucleotide oligomerization domain-like receptors (NLRs, also called NACHT, LRR, and PYD domain proteins) and cytosolic DNA sensors such as DNA-dependent activator of IFN-regulatory factors (DAI), IFN-inducible factor 16 (IFI16), Ku70, stimulator of IFN genes (STING), and cyclic GMP-AMP (cGAMP) synthase (cGAS) [29,30]. Similar to other retroviruses, HTLV-1 produces several forms of nucleic acid including RNA, RNA/DNA intermediates, ssDNA intermediates, and dsDNA during its life cycle and provides opportunities for sensing via different immune sensors to recognize viral invasion and trigger immune responses (Figure 1) [31].

### 3.1. TLRs

TLR3 is widely expressed in the endosomal compartment of innate immune cells and recognizes retroviral double-stranded RNA, a viral replication intermediate, to trigger antiviral activities through secreting IFNs and inflammatory cytokines [32]. Single nucleotide polymorphisms (SNPs) in the TLR3 promoter region potentially alter gene expression and modulation of TLR3 activity for appropriate antiviral signaling. Intriguingly, Habibabadi et al. showed the TLR3rs3775296 SNP was significantly linked with HTLV-1 infection in an HTLV-1-endemic area of Iran and potentially acts as a protective factor against HTLV-1 infection [33].

The immune response triggered by professional ‘sentinel’ or plasmacytoid dendritic cells (pDCs) is largely induced through TLR7- or TLR9-mediated recognition of RNA from retroviruses [34,35]. Colisson et al. demonstrated that cell-free HTLV-1 virions triggered immune response in pDCs in vitro and promoted the secretion of massive levels of IFN-α in a TLR7-dependent manner [36]. Moreover, HTLV-1 infection drives dendritic cell maturation via CD40, CD83, and CD86 expression and can convert pDCs into killer plasmacytoid dendritic cells (IKpDCs) for the induction of cytotoxic activity mediated by TRAIL [36]. Nevertheless, cell-free viruses are poorly infectious and mostly undetectable in the plasma of HTLV-1-infected individuals, raising fundamental questions with regard to in vivo infection and activation of pDCs by cell-free HTLV-1 virions in the absence of cell-to-cell transmission [37,38]. Furthermore, ex vivo pDCs from HTLV-1-infected patients exhibited diminished IFN-α production, indicating an immune evasion strategy by HTLV-1 to inhibit IFN-α in pDCs [39]. 

### 3.2. IFI16

IFI16 is a critical intracellular DNA sensor that interacts with dsDNA through direct binding and is characterized as an IFN-inducible protein in antiviral immune responses [40]. IFI16 is also involved in DNA damage and regulation of cell cycle checkpoints [41]. The antiviral immune response function of IFI16 was first identified during herpes simplex virus type-1 (HSV-1) infection and was also investigated in other DNA virus infections including human cytomegalovirus (HCMV), Kaposi’s sarcoma-associated herpes virus (KSHV), and Epstein–Barr virus (EBV) [42,43,44,45]. IFI16 forms a scaffold with viral DNA to recruit the endoplasmic reticulum (ER) adaptor STING and TANK-binding kinase 1 (TBK1) for the activation of IRF3 and NF-κB signaling to induce IFN-β and initiate antiviral activity [42,46]. HTLV-1 infection induces IFI16 expression, and IFI16 interacts with the reverse transcription intermediate (RTI) ssDNA90 [47]. Of note, IFI16 enhances HTLV-1-induced innate immune responses through increased production of IFN-β, TNF-α, CCL5, and CXCL10 and promoting phosphorylation of IRF3 and p65 in a STING-dependent manner [47]. IFI16 also limits HTLV-1 replication through inhibition of HTLV-1 protein expression [47].

### 3.3. STING

STING is an ER-localized adaptor that plays a critical role in the cGAS DNA sensing pathway. cGAS senses viral or host dsDNAs and generates the cyclic dinucleotide 2′3′-cGAMP (cyclic GMP-AMP) that binds to and activates STING. STING then traffics to the Golgi where it recruits TBK1 for downstream activation of IRF3 and NF-κB to induce type I IFNs and proinflammatory cytokines to limit viral replication [48,49]. STING promotes IRF3-mediated antiviral signaling in response to HTLV-1 RTI ssDNA90 and stimulates type I IFN production [50]. STING also triggers an apoptotic response in HTLV-1-infected monocytes, leading to abortive infection [50]. 

### 3.4. Ku70

Ku70 is well studied for its crucial role in the dsDNA break repair pathway through non-homologous end joining in a complex with Ku80 and the catalytic subunit DNA-PKcs [51,52]. Recent evidence revealed its multifunctional roles in several other cellular processes such as apoptosis, aging and HIV-1 replication, and may also function as a DNA sensor through interaction with dsDNA from several viruses including HSV-1 and HBV [53,54,55,56,57]. Ku70 triggers type III IFNs through a STING-TBK1-IRF3, IRF1, and IRF7 signaling pathway. Ku70 is mainly localized in the nucleus and, upon DNA stimulation or viral infection, is exported from the nucleus to the cytoplasm to stimulate STING-mediated innate immune responses [58]. During HBV infection, Ku70, in cooperation with Ku80, recognizes HBV DNA followed by DNA-PKcs and PARP1-mediated stimulation of hepatitis-associated chemokine secretion [57]. Intriguingly, the level of Ku70 can be induced upon HTLV-1 detection through binding with HTLV-1 RTI ssDNA90, which then triggers antiviral responses through increased phosphorylation of IRF3 and p65, leading to secretion of IFN-β, TNF-α, and ISG56 in a STING-dependent manner [55]. The elevated expression of Ku70 was also associated with reduced expression of HTLV-1 proteins, indicating impaired HTLV-1 replication [55]. Recent studies also reported the critical role of DNA-PK as a DNA sensor for viral infection that triggers innate immune responses, and the Ku70/Ku80 complex enhances the sensing capability of DNA-PK for dsDNA [59,60]. During the HTLV-1 replication cycle, HTLV-1 linear dsDNA is trafficked to the nucleus, but only a fraction of the DNA integrates into the host genome [31]; thus, it is plausible that Ku70, together with DNA-PK, may also sense HTLV-1 dsDNA to trigger innate immunity. Additional investigations are warranted to shed light on the in-depth mechanisms of the DNA-PK complex in DNA sensing during HTLV-1 infection. 

## 4. HTLV-1 Restriction Factors

**Figure 2:**
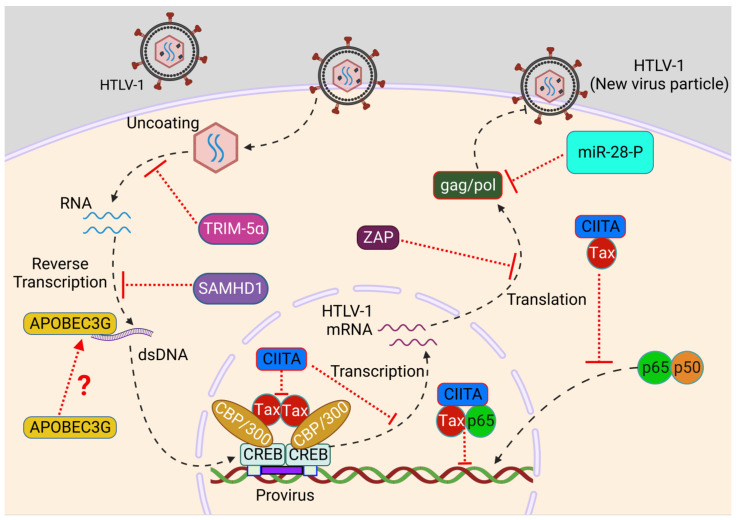
Role of restriction factors during HTLV-1 infection. After HTLV-1 entry, the uncoating of virus results in the release of dimeric RNA viral genome into the cytoplasm that undergoes reverse transcription to form double-stranded DNA. TRIM5α targets the uncoating of virus through inducing degradation of the viral capsid, while SAMHD1 blocks reverse transcription of HTLV-1 RNA to restrict viral replication. APOBEC3G may suppress HTLV-1 replication through the incorporation of G-to-A mutations. CIITA blocks Tax-mediated LTR activation and NF-κB activation to prevent HTLV-1 long-term persistence. ZAP induces viral mRNA degradation to block viral protein translation and miR-28-P targets Gag/Pol genomic viral mRNA to restrict HTLV-1 de novo infection. Created with BioRender.com.

Innate and adaptive immunity cooperate in multiple ways to counteract viral infection. In addition, several host intrinsic cellular proteins termed restriction factors (RFs) contribute to the frontline defense to impede viral infections. Restriction factors are either constitutively expressed or induced by mediators of innate immunity such as type I IFN (interferon-stimulated genes or ISGs) to recognize and inhibit viral replication at multiple steps of the viral infection cycle, in a cell-autonomous manner [61,62]. RFs were originally discovered in the HIV research field, and since then, several RFs have been identified, belonging to diverse protein families that drive antiviral responses through multiple mechanisms [62,63,64]. Recent studies, described below, have highlighted the role of several restriction factors involved in the protection against HTLV-1 infection and their modes of action (Figure 2).

### 4.1. APOBEC3 

Apolipoprotein B mRNA-editing enzyme catalytic polypeptide-like 3 (APOBEC3) catalyzes the deamination of cytidine to uridine in single-stranded DNA substrates resulting in G-to-A mutations [65]. APOBEC3, especially A3G, also plays a critical role in inhibiting retroviral and DNA virus replication [66,67]. A3G is incorporated into newly synthesized HIV-1 viral particles, delivered into infected cells along with the viral genome, and induces G-to-A mutations during reverse transcription to hamper viral genome integrity. However, HIV-1 counteracts the inhibitory function of A3G through the Vif protein, triggering its degradation or preventing its packaging into new viral particles [68]. Surprisingly, HTLV-1 infection seems to be resistant to APOBEC3 proteins [69]. During the HTLV-1 infection cycle, although A3G is encapsidated into HTLV-1 particles, A3G-driven G-to-A mutations were not detected in the proviruses of HTLV-1-infected individuals [70,71]. Although in vivo expression of A3G was elevated in patients with HTLV-1, no correlation was established with clinical status or proviral load [72,73]. However, Sasada et al. demonstrated that overexpressed and endogenous A3G blocked HTLV-1 infection [74]. This paradox could be explained by levels of A3G packaging into HTLV-1 viral particles due to elements in the C-terminus of Gag [71]. Another study involving other proteins of the APOBEC3 family revealed the inhibitory effects of A3A, A3B, and A3H haplotype 2 (A3H hapII) during HTLV-1 infection, indicating HTLV-1 could be a potential target of multiple APOBEC3 family proteins [75]. More investigations are warranted for an in-depth understanding of HTLV-1 resistance mechanisms towards A3G and potential exploitation of APOBEC3 family proteins by the HTLV-1 virus for successful immune evasion.

### 4.2. SAMHD1

Although HTLV-1 predominantly infects T-cells, myeloid cells are also potential targets of HTLV-1 infection [27,76,77]. The cellular SAM domain HD domain-containing protein 1 (SAMHD1) is highly expressed in myeloid cells and restricts HIV-1 replication through targeting pre-integration steps and prevents productive DNA synthesis through depleting the dNTP pool [78,79]. However, the antiviral function of SAMHD1 is counteracted by HIV-1 Vpx to prevent antiviral immune responses [80]. The role of SAMHD1 during HTLV-1 infection is highly debated. Infection of primary monocytes with HTLV-1 leads to SAMHD1-mediated apoptosis, and SAMHD1 also blocks HTLV-1 infection through RTI-stimulated STING-IRF3-BAX-driven apoptosis. SAMHD1 also depletes the endogenous dNTP pool to block reverse transcription [50]. Accordingly, deficiency of SAMHD1 and supplementation with exogenous dNTPs rescues reverse transcription without induction of apoptosis, suggesting an antiviral role of SAMHD1 against HTLV-1 infection [50]. In contrast, degradation of SAMHD1 in DCs and macrophages via SIV-Vpx prior to HTLV-1 infection failed to enhance HTLV-1 infection in vitro, suggesting that HTLV-1 is resistant to SAMHD1-mediated restriction [81]. Therefore, further investigations are needed to assess the role of SAMHD1 during HTLV-1 infection.

### 4.3. Tetherin

Tetherin, also known as bone marrow stromal cell antigen 2 or BST-2, functions as a restriction factor through inhibiting the release of viral particles from infected cells; however, HIV-1 Vpu antagonizes the action of tetherin in retaining viral particles at the cell surface through depleting its expression [82,83,84]. Interestingly, HTLV-1 lacks the expression of a viral tetherin antagonist and chronically HTLV-1-infected cells display high levels of tetherin expression, indicating that HTLV-1 viral dissemination is not impacted by the tetherin restriction factor [19]. The inability of tetherin in restricting HTLV-1 infection could be explained through the difference in the mode of infection in HIV-1 and HTLV-1, as HTLV-1 cell-free virions are poorly infectious and HTLV-1 transmission solely depends on cell-to-cell contact. The retention of newly synthesized viruses at the surface of infected cells may actually favor cell-to-cell contact-mediated transmission, suggesting an HTLV-1 immune evasion strategy to counteract the antiviral function of tetherin. Interestingly, tetherin has also been recognized as a component of viral biofilms, supporting the notion that tetherin could be a potential proviral factor during HTLV-1 infection [85].

### 4.4. CIITA

The MHC class II transcriptional activator (CIITA) plays a critical role in triggering adaptive immune responses through efficient control of antigen presentation to CD4+ helper (TH) cells and can also initiate direct antiviral activity through inhibiting viral expression in a cell-autonomous manner [86,87]. CIITA is constitutively expressed in B cells and induced in T-cells upon activation with antigen stimulation [88]. The antiviral function of CIITA was first investigated in the context of HTLV-2 infection, and it was demonstrated that the transactivation function of Tax-2 was inhibited by CIITA to restrict HTLV-2 replication and infection [89]. CIITA also prevents HTLV-1 replication through suppressing Tax-mediated LTR activation by impairment of the interactions between Tax and cyclic AMP-responsive element binding protein (CREB), the cellular coactivators p300/CBP-associated factor (PCAF), and activating transcription factor 1 (ATF1), all essential factors for Tax-mediated LTR activation [90]. Moreover, CIITA interacts with Tax and retains an inactive p50/RelA/IκB complex in the cytoplasm to inhibit Tax-mediated NF-κB activation. Nuclear CIITA also associates with Tax/RelA in nuclear bodies and prevents Tax-dependent activation of NF-κB-responsive genes. Thus, CIITA hinders oncogenic transformation of infected cells through blocking Tax-mediated cytoplasmic and nuclear NF-κB activation, which is crucial for cell survival and persistent infection [91]. Hence, CIITA acts as an endogenous restriction factor impeding Tax-mediated HTLV-1 gene expression and replication, thus preventing viral spread and associated pathogenesis. However, further investigations are necessary to decipher the antiviral role of CIITA in HTLV-1-infected myeloid cells.

### 4.5. ZAP

The CCCH-type zinc finger antiviral protein, also known as ZAP, successfully restricts retroviral infections such as HIV-1, murine leukemia virus (MLV), and avian leukosis virus (ALV) as well as several other viruses including Hepatitis B virus, influenza A virus, and flaviviruses [92,93,94,95,96]. ZAP discriminates between host and viral mRNAs through selectively binding to CpG-rich sequences in viral mRNAs and induces degradation of viral mRNAs to prevent viral gene expression [97]. A recent study has revealed the susceptibility of HTLV-1 to ZAP-mediated suppression, as the HTLV-1 proviral sequence is enriched in CpG sequences. The overexpression of ZAP leads to a reduction in HTLV-1 production in a dose-dependent manner and deficiency of ZAP results in an increase in virus production, suggesting an antiviral effect of ZAP in restricting HTLV-1 expression and production [98]. 

### 4.6. TRIM Family

The tripartite motif (TRIM) family comprises several interferon-induced proteins that carry out key roles in restricting viral infections [99]. TRIM5α targets HIV-1 replication at an early stage through binding to the viral capsid and triggering ubiquitin-dependent degradation, leading to impairment of virus uncoating [100,101]. HIV-1 is unable to counteract the antiviral function of TRIM5α, making it a suitable target for the development of HIV-1 therapeutics. However, the uncoating of the HTLV-1 capsid is poorly investigated and lacks in-depth mechanistic understanding. Unlike HIV-1, there is no evidence of a direct interaction between TRIM5α and HTLV-1. However, a TRIM5α polymorphism has been linked with high proviral loads in PBMCs from HAM/TSP patients, indicating an antiviral role of TRIM5α in restricting HTLV-1 infection [102]. Another study using PBMCs from asymptomatic and HAM/TSP patients confirmed an antiviral function of TRIM5α for HTLV-1 through transcriptomic analysis and showed a negative correlation between TRIM5α and the proviral load of HTLV-1, assessing the level of expression of Tax and HBZ [103]. 

Another TRIM family member, TRIM19 (also known as promyelocytic leukemia protein (PML)), also functions as a restriction factor against HTLV-1 infection [104]. Nuclear bodies containing Tax undergo PML-dependent hyper-SUMOylation via small ubiquitin-like modifier (SUMO)2/3 and ubiquitination via RNF4, resulting in proteasomal degradation of Tax and apoptosis of HTLV-1-infected cells [104]. Hence, TRIM family proteins could represent promising therapeutic targets as host cellular factors restricting HTLV-1 infection and HTLV-1-mediated pathogenesis.

### 4.7. ADAR1

Adenosine deaminase acting on RNA (ADAR) is a cellular RNA-editing protein involved in post-transcriptional processing of dsRNA and comprises three members: ADAR1, ADAR2, and ADAR3 [105]. Type I interferon induces the expression of isoform ADAR1-p150, which has been implicated in antiviral immune responses. ADAR1 was shown to edit and deplete the hepatitis C viral (HCV) genome to restrict HCV infection [106,107]. However, ADAR1 can also function as a proviral factor for other viruses such as KSHV, HIV, and EBV [108,109]. Mounting evidence supports a proviral role of ADAR1 that is independent of its RNA-editing activity and rather relies on inhibition of protein kinase R (PKR) function [110]. Interestingly, ADAR1 is expressed in both activated peripheral blood lymphocytes and in an HTLV-1 chronically infected T-cell line [111]. However, ADAR1 enhances the susceptibility of target cells to HTLV-1 infection and amplifies viral gene expression and production of infectious viral particles [111]. Thus, ADAR1 acts as a proviral factor independent of its RNA editing activity during HTLV-1 infection, despite being an ISG. Moreover, the proviral effect of ADAR1 is mediated through inhibition of PKR, suggesting an immune evasion strategy of HTLV-1 to mitigate PKR activity via modulating the function and expression of ADAR1 [111]. Interestingly, IFN-α can block HTLV-1 de novo infection via PKR activation [111,112]. 

### 4.8. Micro RNAs

Micro RNAs (miRNAs) can also act as restriction factors to defend against retroviral infections, and recent studies have highlighted the importance of certain miRNAs in HTLV-1-associated disease pathogenesis [113,114,115]. miR-28-3p may contribute to restricting viral infection as its expression was amplified by IFN-α or IFN-γ stimulation [116]. Interestingly, miR-28-3p inhibits HTLV-1 expression and reverse transcription through targeting a sequence located within the viral Gag/Pol genomic viral mRNA during de novo infection [117]. Therefore, elevated levels of miR-28-3p in uninfected cells provide resistance to HTLV-1 infection [117]. High expression levels of miR-155 and miR-146a were also observed in ex vivo ATL cells and HTLV-1-transformed cells [118]. miR-155 potentially targets IRF3 signaling through antagonizing IKKε (also known as IKKi) expression [119,120,121,122]. miR-146a targets and downregulates IRAK1, IRAK2, and TRAF6 to dampen TLR and RLR signaling and block IFN and ISG expression [123,124,125]. Together, miRNAs play complex roles in HTLV-1 infection and disease pathogenesis, and more studies are needed to shed light on the mechanisms of proviral and antiviral miRNAs in HTLV-1 infection.

## 5. Innate Immunity and Inflammation 

Innate immune-mediated inflammation plays a critical role in inhibiting pathogenic viruses. Innate immune receptors trigger the production of pro-inflammatory cytokines and interferons (IFNs), as well as signals that recruit and activate cells that orchestrate inflammation and the induction of adaptive immunity. Likewise, cellular immune responses have been implicated in the control of HTLV-1 infection as well as the development of inflammatory disorders in patients [126]. Members of the NOD-like receptor (NLR) family of cytosolic PRRs activate pro-caspase-1 signaling and secretion of the pro-inflammatory cytokines IL-1β and IL-18 through formation of an inflammasome complex comprising an NLR, the adaptor apoptosis-associated speck-like protein containing a CARD (ASC), and the CARD domain containing pro-caspase-1 [127,128]. NACHT, LRR, and PYD domain-containing protein 3 (NLRP3) recognizes diverse PAMPs and DAMPs generated during viral replication to elicit NLRP3 inflammasome-dependent antiviral immune responses that contribute to viral clearance [129]. HTLV-1 also induces significant IL-1β secretion, indicating its cytoplasmic recognition by inflammasomes [130]. Moreover, Kamada et al. showed that an NLRP3 polymorphism (rs10754558 G/G) was associated with protection against HTLV-1 infection in a northeastern Brazilian population [131]. However, HTLV-1 p12 can suppress inflammasome activation to enhance viral transmission [132]. ATLL cells also secrete TNF𝛼 that can promote inflammation, and a TNF𝛼 polymorphism was linked to increased susceptibility to ATLL development in HTLV-1 carriers [133].

Although the induction of innate immunity and inflammation in response to viral infection is important to control virus replication, several oncogenic viruses, including HTLV-1, modulate inflammatory effector molecules that promote an environment conducive to cancer development. Mounting evidence suggests that acute inflammation is mostly associated with an antipathogenic role, whereas chronic inflammation favors cancer development [134]. Moreover, disruption of the balance between inflammatory and anti-inflammatory responses leads to a loss of tolerance and the development of autoimmunity and inflammatory disorders [135]. HTLV-1 Tax constitutively activates JAK-STAT and NF-κB pathways that promote chronic inflammation and the long-term persistence of proviral clones that can eventually result in leukemogenesis or inflammatory disorders such as HAM/TSP [136,137,138]. However, HBZ suppresses canonical NF-κB signaling as a potential immune evasion mechanism, but it maintains chronic inflammation through NIK-mediated non-canonical NF-κB activation, and also interacts with STAT3 to modulate the IL-10/JAK/STAT signaling pathway for T-cell proliferation [139,140,141]. 

## 6. Innate Immune Evasion by HTLV-1

**Figure 3:**
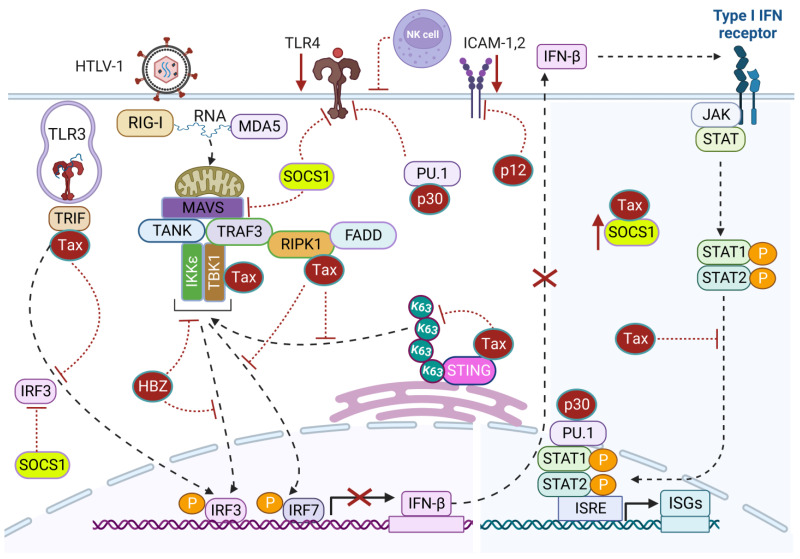
HTLV-1-mediated suppression of immune signaling. HTLV-1 Tax targets and inhibits immune signaling triggered by RNA sensors TLR-3 and RIG-I/MDA5 through interacting with TRIF and RIPK1, respectively, to block IRF3- and IRF7-mediated IFN-β production. Tax also interacts with STING and prevents its K63-linked polyubiquitination to impair TBK1-mediated immune signaling. Tax interacts with and stabilizes SOCS1 expression to block TLR4 and RIG-I signaling. Tax also competes with STAT2 to diminish the production of ISGs via IFN-induced JAK-STAT signaling. HTLV-1 p30 downregulates TLR4 expression through the PU.1 transcription factor and interferes with the production of type I IFN and ISGs. HTLV-1 p12 alters the expression of ICAM-1 and 2 to impair the adherence of NK cells to infected cells and protect against NK cell-mediated cytotoxicity. Created with BioRender.com.

HTLV-1 infection triggers the stimulation of multiple PRRs and activation of immune pathways and ISGs to counteract viral infection at multiple steps during the HTLV-1 replication cycle. To establish successful infection, replication, and persistence, HTLV-1 employs numerous immune evasion strategies to counteract innate immune responses targeting the virus. HTLV-1 targets and manipulates different immune sensors, restriction factors, transcription factors, and downstream signaling cascades for IFN production. These immune escape mechanisms disrupt antiviral immune signaling to prevent host-mediated viral clearance and facilitate HTLV-1 persistence and potential development of HTLV-1-associated pathogenesis. Understanding the underlying immune escape mechanisms of HTLV-1 is essential to design and develop novel antiviral therapeutics. Below, we have highlighted known strategies utilized by HTLV-1 to evade host antiviral defense programs (Figure 3).

### 6.1. Tax

The pX region of the HTLV-1 genome encodes a regulatory protein Tax which acts as a transcriptional activator that promotes viral gene expression through the recruitment of cellular transcription factors such as CREB and CBP/p300 to the viral 5′ LTR promoter [142]. Tax is also an oncoprotein that promotes the clonal proliferation and long-term persistence of proviral clones that may eventually acquire genetic and epigenetic changes that favor clonal outgrowth and leukemogenesis [137]. Tax exerts its pleotropic functions through interacting with a plethora of host proteins regulated via multiple post-translational modifications [143,144,145]. The functional domains of Tax, including a nuclear localization signal (NLS) and nuclear export signal (NES), control the dynamic trafficking of Tax between the cytoplasm and nucleus for activation of NF-κB or viral gene expression [146,147,148]. However, the protein expression of Tax is almost undetectable in ATLL PBMCs, due to genetic or epigenetic changes in the viral genome; many Tax-expressing cells are also eliminated by cytotoxic T-cells (CTLs) due to the strong immunogenic properties of Tax [149,150,151]. Of note, a sporadic burst of Tax expression in a small percentage of infected cells ensures the survival of the whole leukemic cell population [152]. The critical role of Tax in maintaining persistent infection is also accompanied by an array of immune evasion strategies to protect infected cells against immunosurveillance and innate immune sensors. 

Intriguingly, Tax interacts with and inhibits the adaptor TIR-domain-containing adapter-inducing interferon-β (TRIF), likely to impair endosomal sensing of dsRNA through TLR3 signaling and downstream innate immune signaling [153]. Moreover, Tax impedes retinoic acid-inducible gene I (RIG-I) and melanoma differentiation-associated protein 5 (MDA-5)-mediated production of type I IFN and also abrogates Poly(I:C)-induced secretion of type I IFN [153]. Thus, Tax establishes a highly immunosuppressive environment in chronically infected cells. Receptor-interacting serine/threonine-protein kinase 1 (RIPK1) is a downstream mediator of the RIG-I/MDA-5 pathway, and Tax interacts with RIPK1 to impair IFN-α induction [153]. IRF7 (interferon regulatory factor 7) is considered the master regulator of IFN-α production, and Tax can bind to RIPK1 and disrupt RIPK1/IRF7 interaction to block IRF7 activation [153]. Mechanistically, Tax interacts with the RIP homotypic interaction motif (RHIM) domains of TRIF and RIPK1 to suppress IRF7 activity.

The cGAS-STING pathway recognizes dsDNA and triggers innate immune responses through IRF3-mediated IFN-β production. HTLV-1 circumvents the antiviral action of type I IFN through Tax-driven suppression of IRF3 activity [154]. Tax interacts with the kinase TBK1 and suppresses TBK1-mediated phosphorylation of IRF3 [154]. However, the impairment of IFN-β production is independent of Tax-mediated CREB or NF-κB activation [154]. Another study suggested that Tax may directly interact with STING to blunt its K63-linked polyubiquitination and downstream TBK1 recruitment and IFN-β induction [155]. STING deficiency enhances HTLV-1 protein expression, suggesting that STING exerts antiviral activity against HTLV-1 [155]. 

IFNs trigger ISG expression through Janus kinase (JAK)-dependent phosphorylation of signal transducer and activator of transcription (STAT) 1 and STAT2 to amplify IFN-mediated antiviral immune responses and block viral replication at multiple steps. Tax prevents IFN-induced JAK-STAT signaling through competing with STAT2 for CBP/p300 coactivators [156]. Moreover, HTLV-1 exploits suppressor of cytokine signaling-1 (SOCS1) function to dampen the antiviral IFN action for efficient HTLV-1 replication. SOCS1 facilitates IRF3 proteasomal degradation through K48-linked polyubiquitination leading to impaired IFN-β secretion and increased HTLV-1 mRNA synthesis [157]. Tax enhances SOCS1 expression through NF-κB-dependent transcriptional activation and also through directly interacting with and increasing SOCS1 stability [158]. Tax-mediated inhibition of RIG-I-dependent antiviral signaling may be dependent on SOCS1 expression [158]. SOCS1 can also inhibit TLR4 signaling through promoting Mal ubiquitination and degradation [159]; therefore, HTLV-1 may suppress TLR4 signaling through SOCS1, potentially establishing an immunosuppressive state with increased susceptibility to secondary infections. Thus, HTLV-1 utilizes multiple mechanisms to evade IFN-mediated antiviral innate immune responses for persistent infection. Although HTLV-1-transformed cell lines such as MT-2 are resistant to type I IFN, which has no effect on HTLV-1 gene expression, IL-2-dependent HTLV-1-infected cell lines with low Tax expression exhibit sensitivity to IFN-α [160,161]. Likewise, PBMCs from HTLV-1-infected individuals are sensitive to IFN-α as Tax protein expression is low or absent in freshly cultured ATLL cells [162,163]. Thus, the efficacy of Tax evasion of immune responses through a blockade of type I IFN is unknown in vivo owing to its low level of expression and genetic/epigenetic silencing in ATLL.

### 6.2. HBZ

The minus strand of the HTLV-1 provirus encodes a bZIP (basic leucine zipper factor) nuclear factor, termed HBZ. Unlike Tax, HBZ is steadily expressed at low levels in vivo because of an intact 3′ LTR and the absence of abortive epigenetic or genetic changes; HBZ promotes the persistent proliferation of HTLV-1-infected T-cells [164]. Interestingly, HBZ is predominantly localized inside the nucleus in cells from HAM/TSP patients and asymptomatic carriers, whereas HBZ is found in the cytoplasm in cells from ATLL patients, indicating its distinct functions in two different diseases [165]. HBZ is well known for its antagonistic actions on Tax functions such as Tax-mediated HTLV-1 transcription via blocking the binding of CREB and the Tax-responsive element (TRE) [166]. However, HBZ utilizes distinct cellular signaling pathways for the uncontrolled proliferation of infected cells, such as Wnt signaling and activation of human telomerase reverse transcriptase (hTERT) [167,168,169]. In contrast to Tax, HBZ is poorly immunogenic and anti-HBZ antibodies are hardly detected in HTLV-1-infected individuals, suggesting an effective immune evasion strategy of HTLV-1 through dampening the expression of Tax to maintain the long-term persistence of HTLV-1-infected cells [170]. HBZ enhances FOXP3 expression and TGF-β signaling to convert infected T-cells to Tregs and diminish immune responses to HTLV-1; however, this process can lead to inflammation through labile FOXP3 expression and subsequent IFNγ induction [171,172]. Furthermore, HBZ mitigates canonical NF-κB signaling to inhibit gene expression linked to innate immune responses and to prevent the expression of senescent markers in HTLV-1-infected cells [173,174]. Additionally, HBZ inhibits IFN-β production through suppressing IRF3 activation via TBK1 and IKKε [175]. HBZ was also shown to interact with IRF7 and enhance IFN-α production in vitro [175]. However, caution should be used when interpreting data from overexpression models. More studies with endogenous proteins are needed to better understand immune evasion roles of HBZ in HTLV-1-infected cells. 

### 6.3. p30

The HTLV-1 doubly spliced mRNA containing open reading frame II (orf-II) encodes a nuclear/nucleolar protein, p30, with both transcriptional and post-transcriptional activity. The p30 protein attenuates active HTLV-1 replication and promotes viral latency through retaining the doubly spliced mRNA encoding Tax and Rex proteins in the nucleus and disrupting the CREB-Tax-p300 complex essential for 5′ LTR activation [176,177]. HTLV-1 p30 also inhibits the expression of Stathmin/oncoprotein-18 (Op-18), a cofactor of NF-κB-dependent transactivation, and destabilizes the complex with RelA to impair Tax-induced NF-κB-signaling and mitigate cytotoxicity in HTLV-1-infected cells [178]. Therefore, HTLV-1 potentially evades innate immune recognition through controlled expression of p30 to maintain persistent infection. Notably, HTLV-1 infects monocytes/macrophages, which are robust producers of IFNs, but their roles in HTLV-1-associated pathogenesis are poorly understood. TLR4 signaling is critical for dendritic cell maturation and has been linked with both innate and adaptive immune responses. Interestingly, p30 directly interacts with the PU.1 transcription factor and downregulates TLR4 surface expression [179]. Subsequently, p30 impairs the expression of proinflammatory cytokines such as MCP1, TNF-α, and IL-8, and promotes the release of the anti-inflammatory cytokine IL-10 from macrophages upon stimulation with lipopolysaccharide (LPS) [179]. This experimental evidence supports the high-level expression of IL-10 found in the plasma of ATLL patients and HTLV-1-infected cells in vitro [180,181]. Furthermore, interaction of p30 and PU.1 hinders its recruitment to the promoters of IFN-responsive genes, leading to inhibition of IFN-responsive genes following stimulation with LPS and poly(I:C). Additionally, suppression of IFN-responsive genes and stimulation of SOCS1 leads to decreased phosphorylation of STAT1, leading to impaired stimulation of ISGs, favoring HTLV-1 persistence [157,182]. Furthermore, p30 prevents the elimination of HTLV-1-infected cells from immunosurveillance through limiting uncontrolled division and deregulating cell cycle checkpoints [183,184]. These collective immune evasion strategies of HTLV-1 via p30 and other viral genes may contribute to the severe immunodeficiency observed in ATLL patients, rendering them susceptible to other malignancies and opportunistic infections.

### 6.4. p12

HTLV-1 open reading frame I (orf-I) encodes a small hydrophobic protein, p12, which can be further processed into p8 through proteolytic cleavage. p12/p8 are not essential for HTLV-1 replication and transformation of infected cells; however, the binding of p12/p8 to IL-2R results in increased phosphorylation of STAT5 that allows T-cell proliferation in the absence of IL-2 and with suboptimal antigen stimulation, thus providing a growth advantage for HTLV-1-infected T-cells [185]. p12 is well known for its immune evasion roles against HTLV-1 antigen-specific cytotoxic T lymphocyte (CTL)-mediated immune surveillance through downregulation of the expression of major histocompatibility complex 1 (MHC-1) on infected cells to avoid their immune recognition and destruction [186,187]. The detection of singly spliced mRNA encoding p12/p8 ex vivo in HTLV-1-infected T-cells and macrophages signifies the critical role of p12 for establishment and maintenance of HTLV-1-infected cells [188,189]. HTLV-1-infected cells expressing low levels of MHC-1 are major targets of NK cells, and p12 also protects infected cells against natural killer (NK)-mediated destruction through downregulation of intercellular adhesion molecule 1 and 2 (ICAM-1, -2), essential for establishing an immune synapse between NK cells and target cells [190]. Thus, p12 impairs the adherence of NK cells to protect infected cells against autologous NK cell-mediated cytotoxicity [190]. In contrast, transformed HTLV-1 cell lines expressing Tax display high levels of ICAM-1 expression; however, ICAM-1 was found to be downregulated in several ATLL cell lines, including primary CD4+ T-cells infected with HTLV-1 [191].

## 7. Concluding Remarks and Future Prospective

HTLV-1 infection is prevalent in different parts of the world with increasing spread of HTLV-1 to non-endemic areas. Although a minority of infected individuals develop HTLV-1-associated diseases later in life, immunosuppression commonly occurs in ATLL which allows for opportunistic infections. There is clearly an unmet need for effective HTLV-1 vaccines, antivirals, and novel therapeutic approaches for ATLL and HAM/TSP. The major obstacle in designing vaccines and effective antivirals stems from an inadequate understanding of the early events of HTLV-1 infection and the host response to infection. Additionally, immune evasion strategies of HTLV-1 and its interactions with the innate immune system are not well understood. Interestingly, HTLV-1 does not express any specific proteins dedicated for immune evasion seen with other retroviruses; also, the functional roles of HTLV-1-encoded proteins in immune modulation only are partially explored. Thus, further studies focusing on the role of HTLV-1-encoded structural proteins along with regulatory or accessory proteins may provide novel therapeutic targets for viral intervention. Of note, targeting molecular events to counteract host-mediated antiviral immunity during the progression from asymptomatic infection to ATLL or HAM/TSP may provide promising strategies to develop novel antivirals against HTLV-1 infection. Several innate immune sensors have been reported to interact with HTLV-1 RNA or DNA at multiple stages of the viral life cycle in different cell types. Interestingly, HTLV-1 infection in vitro involves broad tropism with restricted viral replication in certain cell types in vivo, thus raising the possibility of specific restriction factors or immune mediators triggering IFN production for the prevention of productive replication to generate mature virions. Likewise, cell types susceptible to HTLV-1 infection display minimal or undetectable IFN levels, implying successful immune evasion by HTLV-1 to induce clonal proliferation for long-term persistence. Restriction factors with antiviral action against HTLV-1 are very limited as the majority of investigations are inspired by the HIV field. However, the structural and functional similarity of Tax with foamy virus transactivator protein, Tas, may be instructive in discovering new restriction factors such as TRIM28 and Pirh2 that restrict foamy virus replication through proteasomal degradation of Tas [192,193]. The antiviral action of restriction factors like CIITA and ZAP, targeting both HTLV-1 replication and oncogenesis, could aid in designing molecular targets for the prevention of ATLL. The regulatory proteins of HTLV-1, such as Tax, interfere with type I IFN production for the protection of infected cells; thus, targeting the expression of specific viral proteins with antiviral factors may represent a new approach in inhibiting HTLV-1 clonal proliferation along with increasing susceptibility to IFN-mediated or NK cell-mediated immune killing. Intriguingly, studies involving the use of histone deacetylase (HDAC) inhibitors showed favorable outcomes in treating HTLV-1-associated diseases through reversing latency via boosting Tax expression and enhancing viral replication in T-cells [194]. This approach would increase viral gene expression and replication, thereby exposing infected cells to immune surveillance and leading to the destruction of infected cells via a kick-and-kill approach [194,195]. 

In conclusion, increased mechanistic insight into HTLV-1-triggered immune responses during early infection and the corresponding viral immune evasion strategies may inform new approaches to modulate antiviral innate pathways and restrict the proviral load and possibly prevent HTLV-1-associated diseases. 

## Data Availability

Not applicable.
